# Sex differences in foraging ecology of the chick rearing Brünnich’s Guillemots (*Uria lomvia*) breeding in a High Arctic colony

**DOI:** 10.1038/s41598-026-36586-z

**Published:** 2026-01-20

**Authors:** Karolina Cieślińska, Dorota Kidawa, Lech Marek Iliszko, Michał Goc, Lech Stempniewicz, Dariusz Jakubas

**Affiliations:** 1 Department of Vertebrate Ecology and Zoology, Faculty of Biology, University of Gdańsk, Ul. Wita Stwosza 59, 80-308 Gdańsk, Poland; 2 Institute of Oceanology Polish Academy of Sciences, Ul. Powstańców Warszawy 55, 81-712 Sopot, Poland

**Keywords:** Alcid, Foraging niche, GPS-tracking, Monomorphism, Parental investment, Remote sensing, Ecology, Ecology, Zoology

## Abstract

Some seabirds, such as alcids exhibit sexual monomorphism, often displaying intersexual variation in parental investment and breeding ecology. In the Brünnich’s Guillemot (*Uria lomvia*), both sexes contribute to incubation and chick rearing. However, it has been found that females provide more meals to chicks older than two weeks, while males spend more time defending the nest and exclusively take care of fledglings after leaving the colony. In this study combining GPS-tracking and remote sensing, we investigated sex-specific foraging ecology of the chick-rearing Brünnich’s Guillemots breeding in the High Arctic (where sex differences are poorly recognized). We found that although both sexes performed foraging trips of similar characteristics, males foraged significantly closer to the colony (mean ± SD: 41.8 ± 23.11 km) in shelf zone with optimal foraging conditions (low sea surface temperature) compared to females exploring further located suboptimal foraging areas (high sea surface temperature, greater depths) (54.2 ± 20.85 km). By utilising more diverse habitats, females exploited a broader foraging habitat niche (defined by sea surface temperature, sea depth, and distance from the colony) than males. This study illustrates how females and males of monomorphic seabird species may adopt different foraging strategies to balance their investment during the chick-rearing period.

## Introduction

Patterns of parental investment in seabirds are shaped by a trade-off between the costs and benefits of allocating resources to offspring care. In many marine bird species, parental duties can be shared approximately equally between the sexes – for example in penguins *Spheniscidae* spp.^1^, Black-legged Kittiwakes (*Rissa tridactyla*)^2^, and Northern Gannet (*Morus basanus*)^3^. The Alcini tribe of the Alcidae family, including Little Auk (*Alle alle*)^4^), Brünnich’s Guillemot (*Uria lomvia*)^5–7^), Common Guillemot (*Uria aalge*)^8^), and Razorbill (*Alca torda*)^9^ is characterised by transition from bi-parental to uni-parental care in which the male parent provides care for the fledgling alone, completing chick-rearing away from the breeding colony.

Variation in parental care in this group of seabirds is often related to sexual dimorphism in body size, mate attraction (sexual selection), or nest site defence^10^. Differences in body size, social dominance, territoriality, or predation vulnerability between sexes helps to minimize the risks of intersexual competition for food^10^. However, there are also examples of sex differences in foraging in monomorphic species (e.g.,^3^), suggesting that morphometrics are unlikely to be the primary factor underlying the observed behavioural differences. Sex differences in foraging performance in long-living species like seabirds may be also affected by trade-offs between own condition (investments in lifetime breeding success) and chick survival (investments in current year breeding success) (e.g.^11^). It has been suggested that in the Alcini tribe males invest more in self-provisioning in anticipation of the post-fledging period of paternal-only care at sea, resulting in female-biased provisioning prior to fledging^12,13^.

The Brünnich’s Guillemot (or Thick-billed Murre, hereafter guillemot) is a long-lived colonial seabird with circumpolar distribution nesting on coastal rock cliffs^14^. It belongs to the largest extant member group of the Alcidae family (*Uria* sp.)^15,16^. The guillemot is monogamous, raising a single chick annually on an exposed rocky ledge, which requires constant protection from predators by one of the parents^7^. It is both piscivorous and zooplanktivorous, preferring cold-water (Arctic) prey, with a particular reliance on polar cod (*Boreogadus saida*) and euphausiids^17,18^. The guillemot exhibits only slight sexual dimorphism, with males having more massive skulls and larger bills than females^19,20^. Despite this general morphological similarity, the sexes show behavioural differences in foraging: spatial, by exploiting different depths^5^ or by utilising different areas [e.g., 8] (often with differing properties), and temporally, by foraging at different times during the diurnal cycle (e.g.,^21,22^), or by using different habitat types as the breeding season progresses^8^. These differences between sexes might result from various parental investments or costs. Males in the studied species are significantly more engaged in defence of egg and chicks at breeding site, than females^20,23^ and only they take care of the offspring after they leave the colony^5–7,12^. However, females provided more meals to chicks older than two weeks, while males spent significantly more time attending the breeding site than their female mates^23^.

The mentioned sex differences in foraging ecology have been recorded in the Low Arctic colonies. However, the use of water masses, environmental properties, and niche partitioning by sexes in the High Arctic populations are understudied (to our knowledge only two studies on sex differences in foraging ecology of guillemot have been performed in the High Arctic –^5,24^). To fill this gap in knowledge, in this study combining GPS-tracking and remote sensing, we investigated sex-specific foraging ecology of the chick-rearing guillemots breeding in the High Arctic colony on Spitsbergen. Additionally, given possible temporal prey depletion effect in the studied population^25^, we investigated temporal (intra-seasonal) variability of female and male foraging ecology as the chick-rearing season progressed. We focused on characteristics of foraging trips (duration, total distance travelled and maximal range of foraging trips), and foraging areas (sea surface temperature [hereafter SST], chlorophyll *a* concentration [a proxy of primary production; hereafter CHLA], sea depth [hereafter DEPTH], seabed slope [hereafter SLOPE] and distance between foraging locations and the colony [hereafter COLDIST]). On this basis, we determined the breadth of the foraging habitat niche of GPS-tracked female and male guillemots. A broader foraging niche and home range may indicate a sex has higher foraging plasticity and can exploit a wider range of environments and prey. However, a narrower niche and home range may indicate higher specialization, feeding exclusively in some habitats possibly focusing on a particular type of prey.

We formulated the following predictions:

1. Given a strong intersexual dissimilarity of parental investments in guillemots, we expect sexes to exhibit variability in foraging trip characteristics, and breadth of realized foraging niches. Differences in foraging ecology of females and males could be reflected in exploiting different foraging areas (with various environmental properties) (e.g.,^5^) or at different times of the diurnal cycle (e.g.^21^). Due to the reported lower investment of female guillemots in chick care, manifested, among other things, by lower colony attendance^23^ and lack of fledgling care by them^7^, females may employ a more risky foraging strategy focusing on less predictable prey with higher energy content compared to males focusing on predictable prey but with lower energy content^5^. We therefore expect females to undertake longer foraging trips and utilise a wider range of foraging areas (larger core and home range areas) and habitats (broader foraging habitat niches) than males. In contrast, we expect males to forage more consistently in similar, environmentally stable areas, in line with a strategy focused on risk-averse chick provisioning (narrower foraging niches).
2. Given that in species from the Alcini tribe males are expected to invest more in self-provisioning in anticipation of the post-fledging period of paternal-only care at sea, we expect female-biased provisioning prior to fledging (self-maintenance hypothesis)^12^. We expect this phenomenon to be expressed as a higher number of foraging trips per day and more frequent dives, especially during the late stages of chick-rearing, in females, since they provide more meals to offspring older than two weeks^23^.
3. Given the observed presence of the temporal prey depletion halo effect in the studied colony i.e. depletion of food resources close to the colony as the breeding season progresses due to high intra and inter-specific competition (large colonies of seabirds are present in the studied area)^25^, we expect that at the later stages parental birds will forage further from the colony, and/or dive more frequently to maintain an adequate energy supply for their offspring. We expect that females are more affected by the depletion halo, as they are expected to provide more food at the later stages of chick-rearing, (e.g.,^5,6^).

## Materials and methods

### Study area

**Fig. 1:**
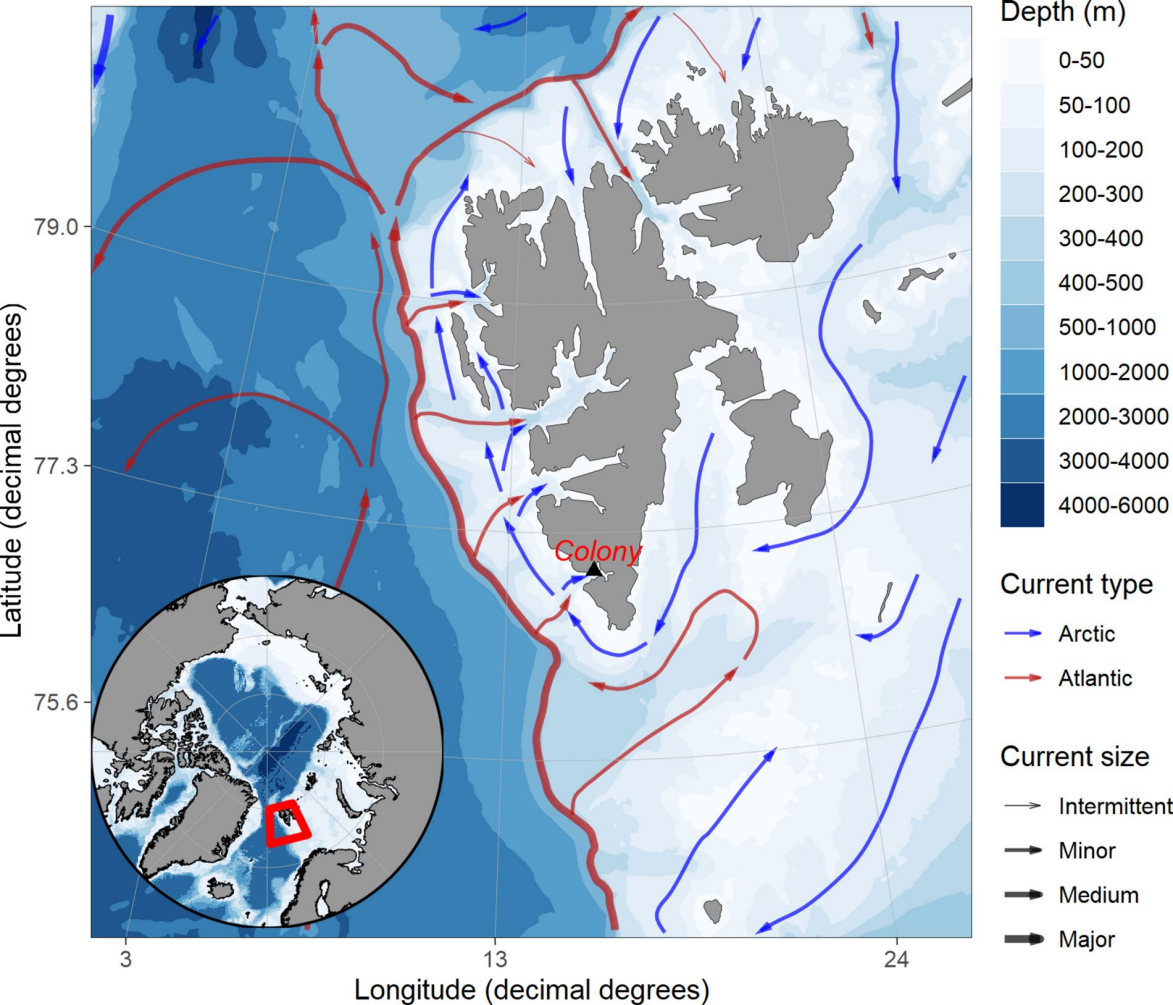
Location of the studied colony of Brünnich’s Guillemots at Gnålodden cliffs in Hornsund (SW Spitsbergen). Blue and red arrows indicate sea currents carrying Arctic-and Atlantic-origin water masses. Maps were generated in the *PlotSvalbard* package (ver 0.9.2.;^39^) in R software ver. 4.5.0 (https://www.r-project.org;^40^). Sea currents distribution according to^41^. Source of bathymetry background map: IBCAO v3.0 500 m RR grid^42^. Base map source: Natural Earth. Free vector and raster map data @ naturalearthdata.com (ver. 4.0.0).

We collected data from the guillemot’s breeding colony located on the Gnålodden cliffs in Hornsund, the southernmost fjord of Spitsbergen, Svalbard^26,27^ (Fig. 1). The size of the investigated colony is estimated at 5,500—10,000 breeding pairs (Norwegian Polar Institute, unpublished database). The shelf zone outside the fjord constitutes as an important foraging area for seabirds (e.g.,^28^) including the studied guillemot^25^. The region is influenced by two water currents, i.e., the coastal Sørkapp Current carrying cold Arctic water and West Spitsbergen Current transporting warm Atlantic water^26,29,30^ (Fig. 1). These two distinct water masses are usually separated by a hydrological front (Arctic or Polar Front) located on the shelf break approx. 250 m isobath^31^. Since these oceanographic features are characterised by high productivity and local prey aggregations^17,32,33^, thermal fronts have been recognized as foraging hotpots for both piscivorous and planktivorous seabirds^17,34–37^, including guillemots^17,38^.

### Fieldwork and GPS-tracking

Fieldwork was conducted during the guillemot’s chick rearing period in July 2016. We captured parent birds on their nest ledges at the colony using a fishing rod fitted with a nylon line terminating in a loop used for safely capturing birds. This capturing approach is widely used in studies concerning cliff-nesting seabirds (see e.g.,^43,44^). We gently put the loop around the neck of the selected bird and after tightening it, we pulled it from the ledge. The captured birds were weighed using a spring balance (with resolution of 10 g) and measured. Standard biometric measurements were collected from captured individuals: wing length, bill length and depth, gonys length, and total head length. We then deployed GPS-loggers (URIA100 & URIA100 Solar 8.5 g; URIA240 & URIA240 Solar 12 g; ALLE100 6 g, ECOTONE, Sopot, Poland) on the birds. The loggers mass ranged from 0.6 to 1.3% of instrumented individuals’ body mass (which ranged from 805 g to 1,065 g) complying with the widely accepted recommendation that tracking devices should not exceed approximately 3% of a bird’s body mass^45^. The GPS loggers were mounted on individuals’ back feathers using stripes of Tesa tape (code 4965, Tesa Tape Inc. Charlotte, NC, USA). Instrumented birds were released after 10–15 min of handling. All GPS-logger equipped guillemots returned to their nesting sites and were later observed at the colony as active breeders. Additionally, the birds were marked with a colour marker to enable us to monitor whether they returned to the nest.

GPS location sampling interval was set to 15 min. Loggers were activated either after the bird’s first contact with salt water (i.e., upon diving) or automatically after 48 h if no diving occurred. Data from GPS-loggers were automatically downloaded to a base station located at the investigated colony. Whenever birds with deployed loggers were within approx. 200 m of the base station, GPS signal acquisition was automatically blocked to save battery life. Reactivation of GPS-loggers occurred always whenever birds left the range of the base station. In total, we deployed loggers on 5 females and 10 males. The duration of GPS data recording per individual ranged from 2 to 22 days (mean ± SD: 9.8 ± 6.06 for females and 12.1 ± 6.72 for males) during the study period (from 8 to 30 July 2016). Results of GPS-tracking of the studied individuals (for both sexes combined) have been published in^25^ investigating inter-annual differences in foraging ecology.

### Sexing of guillemots

Given the very subtle sexual dimorphism in the studied species^19^, we determined the sex of captured individuals using molecular methods. We collected 3–5 contour feathers from each bird and stored them in a cool place until molecular analysis. DNA was extracted using Sherlock AX kit (A&A Biotechnology, Gdynia, Poland). Molecular sexing was performed using the primer pair P2 and P8, specified by the protocol described by^46^. These primers amplify fragments of different length from W chromosome (females only) and Z chromosome (both sexes)^46^. This size difference (length of amplified DNA fragment) is clearly visible during electrophoretic separation of the prepared solution on 3% agarose gel. We successfully sexed 93% (14 out of 15) captured individuals, including 9 males and 5 females.

To sex the single unsexed individual, we performed Linear Discriminant Analysis (LDA) in the *MASS* package (ver. 7.3–60.2;^47,48^) in R software ver. 4.5.0^40^ based on morphological measurements. LDA allows to predict the class of considered observation, based on linear combinations of multiple predictor variables^48^. In this analysis, we used to follow biometric measurements taken from all molecularly sexed individuals (wing length, bill and gonys length, bill depth, and total head length). LDA combined considered measurements into the following model with moderate accuracy (0.643):

$$\begin{aligned} LDA \, = & \, wing*\left( { - 1.08} \right) \, + \, bill \, length*\left( {1.20} \right) \, {-} \, gonys*\left( {0.78} \right) \, \\ & + \, bill \, depth* \, \left( {0.47} \right) \, + \, total \, head \, length*\left( {0.19} \right) \\ \end{aligned}$$

This single molecularly unsexed individual was classified as male with 64.3% fitting accuracy. The efficiency of the discriminant function, based on measured characters in this species, was found to be comparable to the value documented in the literature (0.65;^24^).

### Data analyses and visualizations

All statistical and geospatial analyses were performed in R software ver. 4.5.0^40^. We prepared maps in ArcGIS Pro software ver. 3.2.2 (Redlands, CA, USA: Environmental Systems Research Institute; https://www.esri.com/en-us/arcgis/products/arcgis-pro/overview;^49^) and R software ver. 4.5.0^40^. To pinpoint core and home range area representing the most important foraging areas, we calculated 95% and 50% autocorrelated Kernel Density Estimates (aKDE), respectively)^50,51^, based on foraging locations (stationary locations with slow momentary speed; See details below). aKDE is currently considered as the most proper technique dealing with autocorrelated tracking data^51^. It derives several confidence intervals (estimated value, high-, low-weighted estimates), which properly reflect the limited precision of modelled estimates^51^. We performed aKDE analyses in the *ctmm* package (ver. 1.2.0;^52^).

We compared aKDE area between individuals of both sexes using mixed permutational analysis of variance (MPANOVA) with home or core range area as a response variable, sex as an explanatory variable and bird identity set as a random factor. The number of permutations was set to 5,000 and included the *Rd_kheradPajouh_renaud* resampling method described by^53,54^ implemented in the *permuco* package (ver. 1.1.3;^55^). To evaluate the inter-sex overlap of home and core range areas, we calculated Bhattacharyya’s Affinity (BA) overlap index for both sexes. We calculated BA in the *ctmm* package (ver. 1.2.0;^52^). MPANOVA was used for comparing aKDE sizes between sexes given it’s reduced strict reliance on parametric assumptions and generally small sample size (15 birds). This method increases explanatory power and ensures that the reported *P*-values are valid under fewer distributional constraints^54^.

### Tracking data processing and analyses

To characterise and analyse basic characteristics of foraging trips performed by the GPS-tracked guillemots, we used the *track2KBA* package (ver. 1.1.2;^56^). As a complete trip we considered the one starting and ending within a 1 km radius buffer around the colony and lasting at least 3 h, which is usually observed as a minimal threshold for a trip duration of guillemots (e.g.,^57–59^).

Based on data from GPS-loggers, we calculated several features characterising every foraging trip performed by the studied guillemots:

- (1) maximal range of foraging trip [km], calculated as a straight-line distance between the colony and the most distal GPS location of the particular trip;
- (2) total distance covered [km] during the foraging trip, calculated as a cumulative distance covered between subsequent GPS locations of a single trip;
- (3) trip duration [h], recognized as time spent outside of the colony measured between departure and return of individual to the colony during the single foraging trip;
- (4) distance between the foraging locations and the colony – calculated after pinpointing stationary locations (hereafter called ‘foraging locations’) — the locations with registered momentary speed < 10 km/h; such stationary locations suggest foraging behaviour as low transit speed is commonly considered as an indicator of foraging behaviour of marine predators (e.g.,^60^);
- (5) number of foraging trips per day [*n*] – calculated as the number of trips in particular individuals per 24 h;
- (6) number of dives per trip [*n*] – a summarized number of dives per trip. As a dive we considered a record of contact with seawater recorded by the logger’s conductivity sensor (wet-dry switch, 1 Hz sampling rate). Given deployment of the logger on the back of the birds signals from wet/dry sensor represent dives;
- (7) number of dives per one hour of trip [*n*] – the total number of dives divided by the total duration of all foraging trips combined expressed in hours;
- (8) the number of dives per day [*n*] – the total number of dives divided by the total duration of all foraging trips combined expressed in days;

All mentioned distances were calculated using the *distVincentyEllipsoid* function in the *geosphere* package (ver. 1.5–20;^61^).

### Remote sensed environmental conditions in foraging areas

**Fig. 2:**
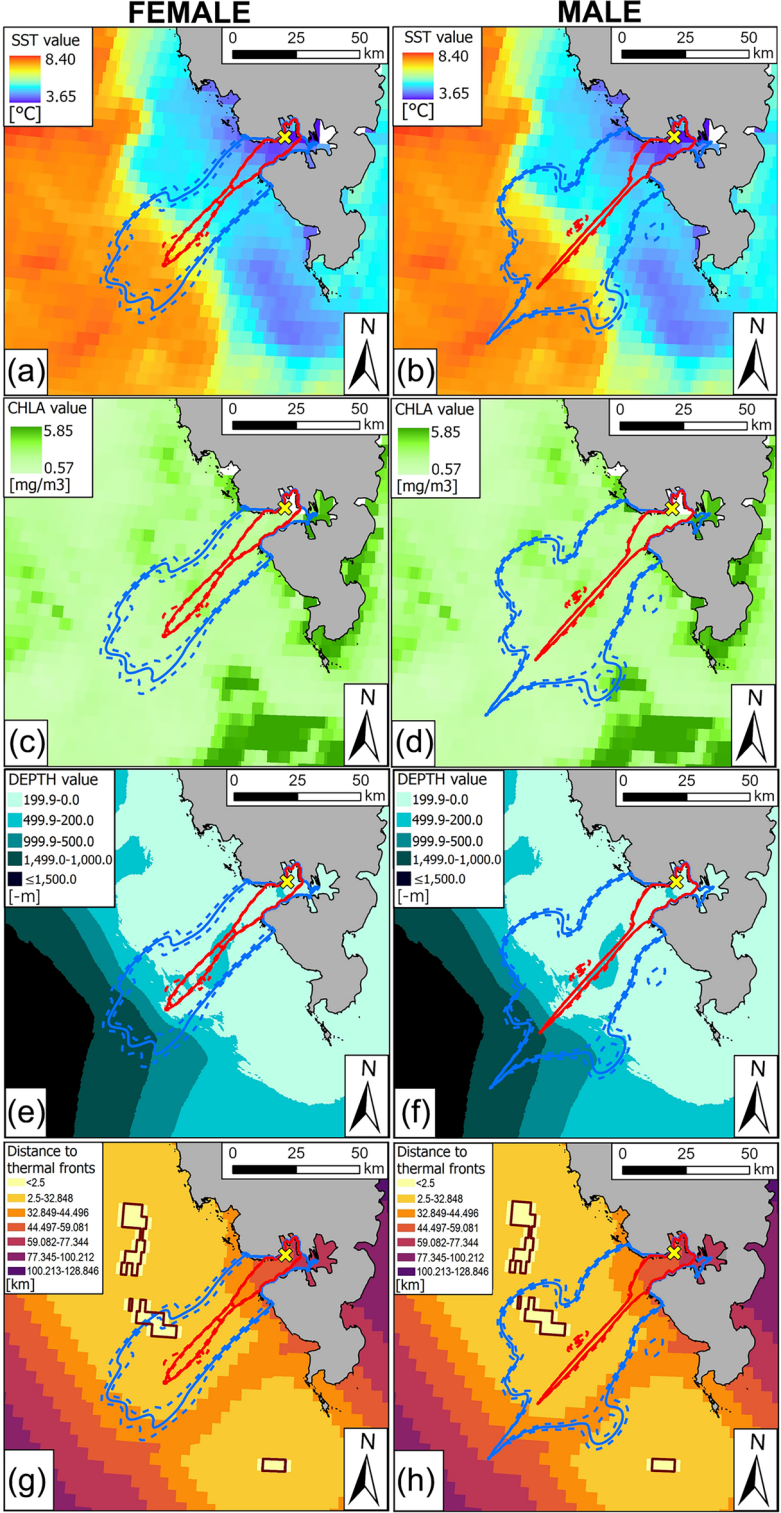
Home and core ranges [Autocorrelated Kernel Density Estimators (aKDE)] of the chick-rearing, GPS-tracked Brünnich’s Guillemots breeding in Hornsund (SW Spitsbergen, Svalbard): females – (**a**;**c**;**e**;**g**) and males – (**b**;**d**;**f**;**h**). Red lines indicate 50% aKDE (core area), while blue lines stand for 95% aKDE (home range). Continuous lines depict estimated aKDE values, while dashed lines indicate high and low estimates. aKDEs calculated based on the foraging locations were visualized on raster with gradient values of environmental variables: sea surface temperature (SST) [°C] (**a**;**b**) and chlorophyll *a* concentration (CHLA) [mg/m^3^] in July 2016, and (**c**;**d**), sea depth (DEPTH) [m] (**e**;**f**), and thermal fronts (brown polygons) [km] (g;h). Euclidean distances to thermal fronts are visualized as reclassified raster map. The first distance class (< 2.5 km) was distinguished arbitrarily, while the others were calculated mathematically on the basis of a geometric series. The yellow x indicates the Gnålloden colony location. White spots in SST and CHLA rasters indicate missing data. Maps were created in ArcGIS Pro software ver. 3.2.2 (Redlands, CA, USA: Environmental Systems Research Institute;^49^). Svalbard land area by the Norwegian Polar Institute (S100 Kartdata)^75^. SST and CHLA datasets were obtained from the Aqua satellite mission^62^ (level 3 monthly mosaics registered by MODIS^63^), DEPTH was derived from the Bathymetric Chart of the Arctic Ocean (IBCAO ver. 5)^71^, the distribution of thermal fronts was identified based on the SST product using an algorithm for identifying oceanic gradients described by^73^ and implemented in the *getGradients* function in the *grec* package (ver. 1.6.1;^74^).

In order to characterise the environmental conditions prevailing at the foraging locations, we used remote sensing data: sea surface temperature (SST) and chlorophyll *a* concentration (CHLA) measurement, recorded by the Moderate-resolution Imaging Spectroradiometer (MODIS) onboard the Aqua satellite^62^. We utilised monthly mosaics from level 3 of data from this mission^63^. Both CHLA (mg/m^3^) and SST (°C) products have a 4 km grid with 0.042 degree vertical and horizontal resolution. We did not use the daily or eight-day mosaic datasets due to data gaps caused by dense cloud cover. SST was included in our analyses since it has been proven to affect the large-scale distribution of the studied species^64,65^ with preference for cold-waters optimal for their prey (e.g.^65–67^,). Areas with high concentrations of CHLA indicate high local productivity zones that serve as hotspot zones for foraging (e.g.,^68^). Finally, we considered bathymetric features (DEPTH, SLOPE) as they are involved in driving stratification regimes and prey aggregations^69,70^. We derived sea depth (DEPTH) at the foraging locations from International Bathymetric Chart of the Arctic Ocean ver. 5 (IBCAO) with spatial resolution of 100 m^71^. The seabed slope (SLOPE) in particular foraging locations was calculated based on sea depth values in 8 neighbouring raster cells of the mentioned IBCAO ver. 5 model in the *terra* package (ver. 1.7–18;^72^). In this study, we also recognized the thermal fronts in the foraging areas based on SST maps. We derived information on the distribution of thermal fronts from the monthly SST imagery described before. Thermal fronts were distinguished using an algorithm for identifying oceanic gradients described by^73^ and implemented in the *getGradients* function in the *grec* package (ver. 1.6.1;^74^). The difference from 0.5 to 1 °C between the raster cells was considered as a thermal front. Due to basing on monthly raster data, we were unable to capture the high real-life variability in the location of thermal fronts. For this reason, we used information on thermal front distribution more for visualization purposes, rather than conducting any specific analyses. Remotely sensed products (e.g. SST and CHLA), the bathymetry model, and the thermal fronts present near the colony are depicted in Fig. 2.

### Factors affecting foraging trips and locations characteristics

To investigate temporal and intersexual differences in number of dives per one hour of trip, the number of dives per day, the number of dives per trip, and the number of trips per day, we computed Generalized Linear Mixed-Effects Models (GLMMs, family = Poisson). The number of dives/trips was set as a response variable, sex (factor variable), day of the year (DOY; a proxy of advancement of the chick rearing period; continuous variable), and sex:DOY interaction as explanatory variables, and bird identity as a random factor. In GLMM with number of dives per one hour of trip, or per 24 h as response variable we set log transformed duration of foraging trips as offset. To investigate temporal and intersexual variation in the colony attendance (time spent daily at the colony), we performed Linear Mixed-Effects Model (LMM) with sex (factor variable), DOY (continuous variable), and sex:DOY interaction as explanatory variables, and bird identity as a random factor. All GLMMs were computed using the *glmer* function while LMMs the *lmer* function in the *lme4* package (ver. 1.1–35.3;^76^).

To investigate temporal prey depletion halo effect, we performed LMMs with foraging trip metrics (maximal trip range, total distance covered, and duration) as response variables, sex (factor variable), day of the year (DOY; a proxy of advancement of the chick rearing period; continuous variable) and sex:DOY interaction as explanatory variables, and bird identity as a random effect. Similarly, we also used LMMs to investigate variation in conditions at the foraging locations with SST, CHLA, DEPTH, SLOPE, and distance from the colony as response variables, and with sex (factor variable), DOY (continuous variable) and sex:DOY interaction as explanatory variables, and bird and trip identity as random effects. LMMs were calculated using the *lmer* function in the *lme4* package (ver. 1.1–35.3;^76^) on the log-transformed values of environmental variables. We visualized results of LMMs in the *ggplot2* package (ver. 3.5.2;^77^).

To select the best GLMM and LMM models^78,79^ we used Akaike’s information criterion for small sample sizes (AICc) using the *dredge* function in the *MuMIn* package (ver. 1.48.11;^80^). We compared the relative performance of the models based on ∆AICc, i.e., the difference between the AIC value of the best model and the AIC value for each of the other models^78^. We considered only models with the lowest AICc.

### Foraging habitat niches

To construct foraging habitat niches of females and males we use only characteristics of foraging locations of GPS-tracked individuals differing significantly between the sexes, i.e., SST, DEPTH, and COLDIST (see tests results in Results). We constructed foraging habitat niches for sexes using the Bayesian Inference Framework in the *nicheROVER* package (ver. 1.1.2;^81,82^). We projected niches as regions of 95% probability at 1,000 runs for higher results accuracy. Finally, we calculated the size of foraging niches, which enabled us to compare their mutual overlap^81^. We compared the breadth of computed foraging niches using two niche regions sizes (95% and 99%)^82^. We compared niche breadth between sexes using Wilcoxon test in the *rstatix* package (ver. 0.7.2;^83^). To depict visualizations of the computed niches, we used two dimensional scatterplots with ellipses standing for ten random projections of the foraging niches in two dimensional perspectives (defined by two variables). We visualized computed niches in the *ggplot2* package (ver. 3.5.2;^77^).

## Results

### Home and core range area of foraging individuals

We found no significant differences in core ranges between females (mean ± SD: 1,514.6 ± 670.75 km^2^) and males (mean ± SD: 1,687.9 ± 1,069.69 km^2^) (MPANOVA, F = 0.11, *P* = 0.75). We also found that home ranges of females (307.6 ± 108.94 km^2^) and males (315.1 ± 239.17 km^2^) were similar (MPANOVA, F = 0.0004, *P* = 0.94) (Fig. 2). Inter-sex overlap in home area was high BA = 0.92 (CI: 0.91–0.92) (Fig. 2 a-h).

### Foraging trips characteristics

During the chick rearing period both sexes of GPS-tracked Brünnich’s Guillemots performed foraging trips with similar characteristics (Table 1 & 2). However, trips characteristics changed with the progress of the chick-rearing period (Table 2). The best LMM model for the maximal trip range and total distance covered included all explanatory variables, while for trip duration – only sex and DOY (Table 2). For every trip characteristic, only DOY had a significant and positive effect (*P* ≤ 0.01), indicating increase of the trip distances and their duration with the progress of the chick rearing period (Table 2). The best GLMM model investigating influence of sex, DOY and their interaction on the number of foraging trips per day was a null model. The next selected models included only DOY that had a non-significant effect on a response variable (*P* = 0.73).

**Table 1:** Characteristics of foraging trips and locations of female and male GPS-tracked Brünnich’s Guillemots (mean ± SD; min–max; median; 25–75% percentiles).

Sex	Foraging trip			Number of	
	Duration [h]	Total Distance covered [km]	Maximal range [km]	GPS-tracked individuals (number of trips)	trips (max per ind.)
Females	12.5 ± 7.26; 3.40–39.13; 11.77; 7.60–13.52	116.1 ± 56.14; 11.08–221.48; 128.13; 69.43–157.02	53.2 ± 25.20; 3.98–100.21; 62.43; 31.85–71.96	5 (35)	15
Males	12.1 ± 7.98; 3.05–48.31; 9.64; 6.83–16.22	86.1 ± 64.32; 7.67–253.28; 72.23; 26.47–144.14	36.4 ± 27.10; 2.37–86.64; 27.25; 10.91–64.27	10 (85)	17

**Table 2:** Results of the highest ranked (selection based on Akaike information criterion) Linear Mixed-Effect Models (LMMs) for factors affecting characteristics of the foraging locations of the GPS-tracked chick rearing Brünnich’s Guillemots breeding in Hornsund (SW Spitsbergen): sex (female, male), day of the year (DOY) and sex:DOY interaction.

Response variables	Predictors	EST	SE	t	CI	*P*	con R; mar R
Foraging trip characteristics							
Duration [h]	sex	9.65082	68.80248	0.14	-126.65; 145.95	0.467	0.24; 0.06
	DOY	0.43255	0.30636	1.412	-0.17, 1.04	**0.011**	
Maximum range [km]	sex	420.389	229.349	1.833	-33.95; 874.73	0.069	0.34; 0.12
	DOY	2.973	1.022	2.908	0.95; 5.00	**0.004**	
	sex:DOY	-2.219	1.170	-1.897	-4.54; 0.10	0.060	
Total distance covered [km]	sex	980.88	537.189	1.826	-83.28; 2,045.05	0.070	0.32; 0.10
	DOY	6.685	2.394	2.792	1.94; 11.43	**0.006**	
	sex:DOY	-5.138	2.740	-1.875	-10.57; 0.29	0.063	
Foraging locations characteristics							
COLDIST [km]	sex	9.52	2.58	3.69	4.46; 14.58	** < 0.001**	0.89; 0.32
	DOY	0.11	0.1	9.55	0.08; 0.13	** < 0.001**	
	sex:DOY	-0.05	0.01	-3.78	-0.08; -0.02	** < 0.001**	
SST [°C]	sex	3.16	0.61	5.22	1.97; 4.35	** < 0.001**	0.84; 0.29
	DOY	0.02	0.003	8.85	0.02; 0.03	** < 0.001**	
	sex:DOY	-0.02	0.003	-5.31	-0.02; -0.01	** < 0.001**	
DEPTH [m]	sex	13.05	1.79	7.31	9.55; 16.55	** < 0.001**	0.72; 0.27
	DOY	0.07	0.007	8.73	0.05; 0.08	** < 0.001**	
	sex:DOY	-0.07	0.009	-7.37	-0.08; -0.05	** < 0.001**	

Response variables: COLDIST – distance to the colony, SST – sea surface temperature, DEPTH – sea depth. Significant terms are bolded. Abbreviations: CI – confidence interval; EST – estimated slope value; SE – standard error; con R – conditional R-squared; mar R – marginal R-squared. Only the highest-ranked LMMs are presented. Significant terms are bolded.

**Fig. 3:**
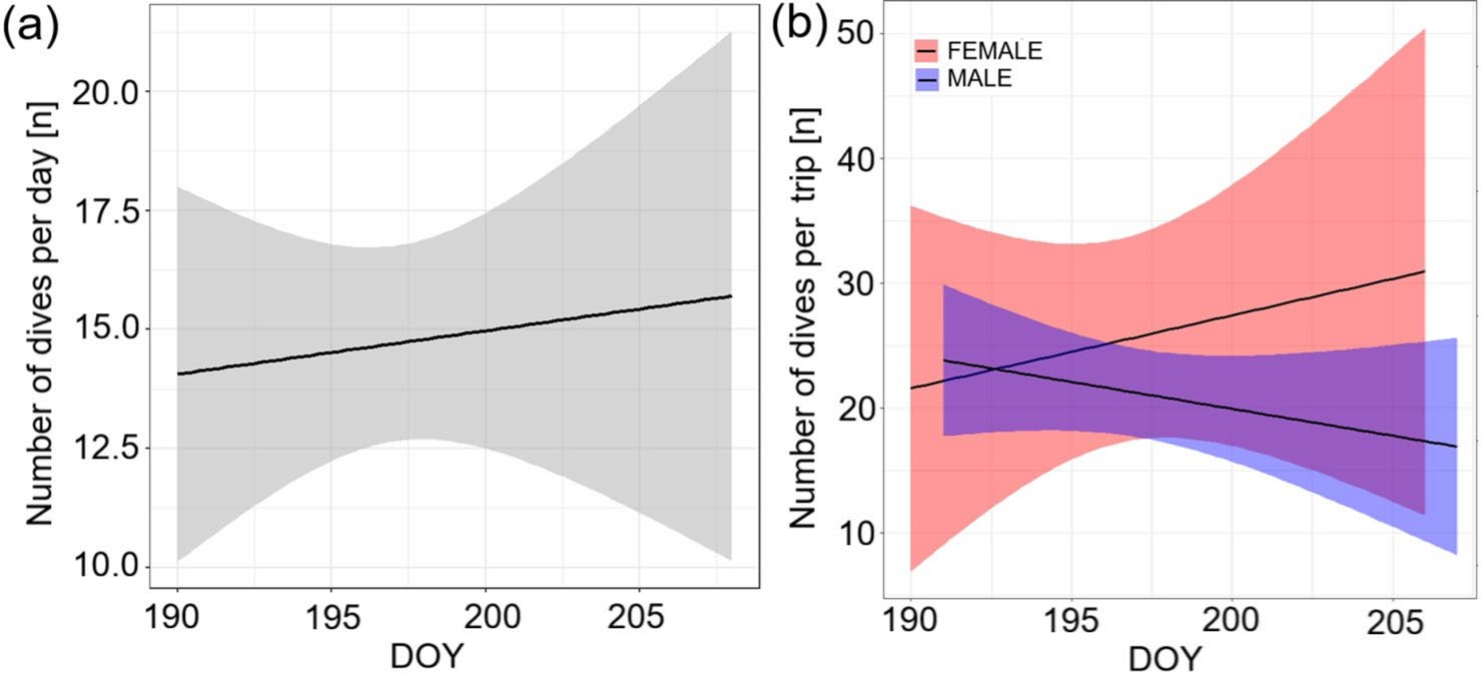
Factors affecting number of dives of the GPS-tracked chick rearing Brünnich’s Guillemots [females (red), males (blue), and both sexes combined (grey)] breeding in Hornsund (SW Spitsbergen). (**a**) – effect of day of the year (DOY) on number of dives per day, (**b**) – effect of the interaction between sex and DOY on the number of dives per trip. Solid lines indicate significant GLMM trends, ribbons around confidence intervals.

The best GLMM model investigating influence of sex, DOY and their interaction on the number of dives per one hour of trip day, included only sex. However, the number of dives per one hour of trip was similar for females (mean ± SD: 1.86 ± 0.83) and males (mean ± SD: 1.88 ± 1.04) (GLMM, z = -1.166, *P* = 0.24). The best GLMM model investigating influence of sex, DOY or their interaction on the number of dives (per day) included all variables, but only effect of DOY was significant. We found that as chick rearing period progressed, birds dived more frequently (GLMM, z = 3.005; *P* = 0.003) (Fig. 3a). There was a tendency for higher number of dives in females (mean ± SD: 17.3 ± 15.48; *n* = 34 trips of 5 ind.) than in males (mean ± SD: 13.7 ± 12.61; *n* = 78 trips of 10 ind.) (GLMM; z = 1.855; *P* = 0.064). There was also a tendency for interaction effect which indicated more rapid temporal increase in number of dives for females than males (GLMM, z = -1.896, *P* = 0.058). The best GLMM model investigating influence of sex, DOY and their interaction on the number of dives per trip included all variables. However, only effect of interaction between sex and DOY was significant (GLMM; z = -2.332; *P* = 0.02). Females increased as males decreased the number dives per trips with the progress of the chick-rearing period (Fig. 3b).

The best LMM model investigating influence of sex, DOY and their interaction on the colony attendance (time spent daily at the colony) included only sex. However, effect of this variable was insignificant (LMM, t = 0.13, *P* = 0.89). Females spent similar amount of time at the colony per 24 h (mean ± SD: 14.32 ± 7.92 h) as males (14.30 ± 7.20 h).

### Foraging locations characteristics

**Fig. 4:**
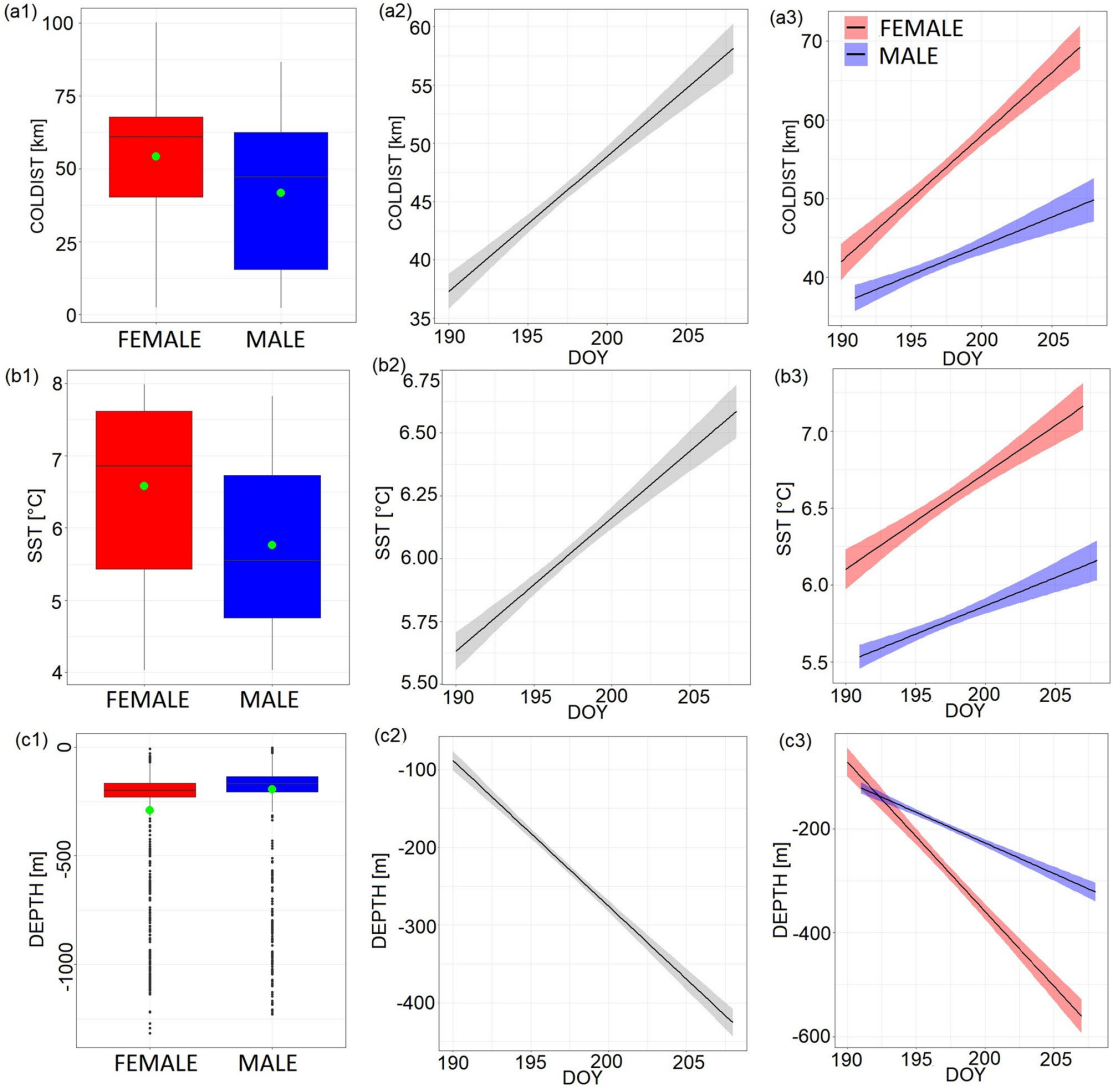
Characteristics of foraging locations utilised by the GPS-tracked chick rearing Brünnich’s Guillemots [females (red), males (blue), both sexes combined (grey)] breeding in Hornsund (SW Spitsbergen) [(**a**) – distance between foraging locations and the colony (COLDIST), (**b**) – sea surface temperature (SST), (**c**) – sea depth (DEPTH)]. (a-c1) – Boxplots for both sexes showing mean (green dot), median (band inside the box), the first (25%) and third (75%) quartile (box), the lowest and the highest values within 1.5 interquartile range (whiskers), and outliers (black dots). (a-c2) – relationship between the studied variables (**a**-**c**) and day of the year (DOY). Solid lines indicate significant LMM trends, ribbons around confidence intervals. (a-c3) – interaction between sex and DOY. Solid lines indicate significant LMM trends, ribbons around confidence intervals.

The best LMM model investigating influence of sex, DOY and their interaction on the distance from the colony included all predictors considered and all of them were significant (Table 2). Foraging locations of females were situated significantly further from the colony (range: 2.5–100.2 km, median 60.7 km, 25–75% percentiles: 40.4–67.8) compared to males (2.3–86.6, 47.2, 15.6–62.5 km) (Fig. 4a1, Table 2,3). Distance from the colony significantly increased with the progress of the chick rearing period (Fig. 4a2, Table 2). Interaction effect was also significant showing more rapid increase of the distance between the foraging locations and the colony with the progress of the chick rearing period in females compared to males (Fig. 4a3, Table 2).

**Table 3:** Environmental conditions in foraging locations of the chick rearing GPS-tracked Brünnich’s Guillemots females and males breeding in Hornsund (SW Spitsbergen): SST – sea surface temperature, CHLA – chlorophyll *a* concentration, DEPTH – sea depth, SLOPE – seabed slope.

Sex		SST [°C]	CHLA [mg/m^3^]	DEPTH [m]	SLOPE [°]
Females	min-max	4.0-8.0	0.8-2.0	1,317.2-7.5	0.02-29.9
	mean±SD	6.6±1.13	1.2±0.20	290.4±261.70	2.2±2.67
	median; IQR	6.9; 2.19	1.2; 0.27	197.9; 64.41	1.3; 2.20
Males	min-max	4.0-7.8	0.9-3.5	1,229.8-1.7	0.01-21.70
	mean±SD	5.8±1.08	1.3±0.28	192.9±155.88	1.7±1.63
	median; IQR	5.5; 1.97	1.3; 0.29	167.0; 71.98	1.2; 1.49

The best LMM models assessing how environmental properties of the foraging sites are affected by sex, DOY and their interaction also included all considered predictors. For SST and DEPTH the effect of all of them was significant (Table 2). Females exploited warmer waters than males (Fig. 4b1, Table 3). All studied individuals regardless of the sex utilised warmer water masses with the progress of the chick rearing period (Fig. 4b2, Table 2). Interaction effect was also significant showing more rapid increase in SST in foraging locations exploited by females with the progress of the chick rearing period (Fig. 4b3, Table 2). The studied females foraged in locations with higher sea depths compared to males (Fig. 4c1, Table 3). Also, as the chick rearing period advanced both sexes exploited areas with greater sea depth (Fig. 4c2, Table 2). Interaction effect was also significant indicating more rapid increase in sea depth in foraging locations exploited by females with the advancement of the chick rearing period (Fig. 4c3, Table 2). The highest ranked LMMs for CHLA and SLOPE were the null models and next in order models included only sex. However, sex in those models had a non-significant effect on response variables (all *P* > 0.1) and are not presented.

### Breadth of environmental conditions niches of sexes

**Fig. 5:**
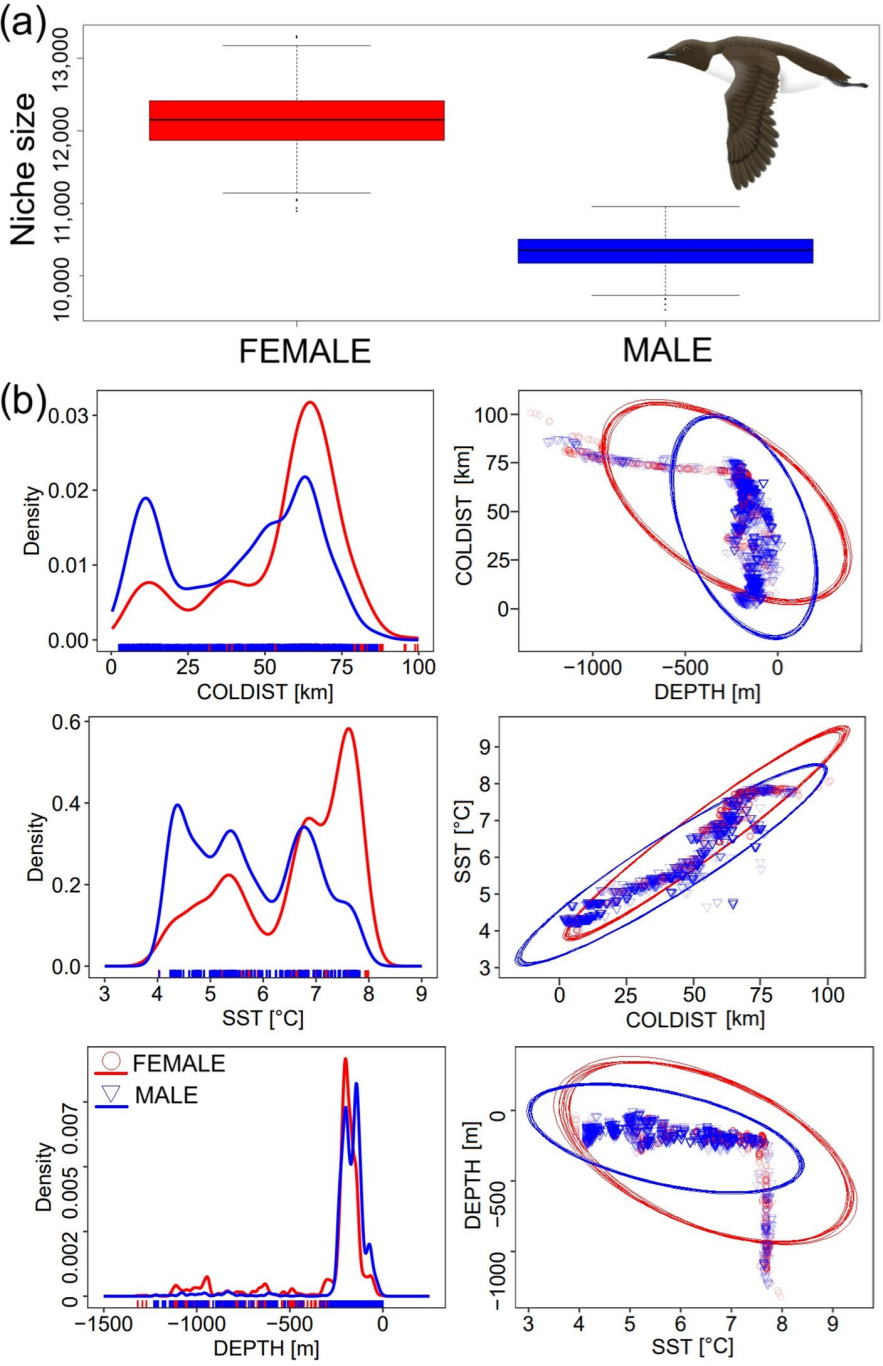
(**a**) Foraging habitat niche breadth of the chick rearing GPS-tracked Brünnich’s Guillemots breeding in Hornsund (SW Spitsbergen). (**b**) projections of foraging habitat niches (described by SST (sea surface temperature), DEPTH (sea depth), and COLDIST (distance between foraging locations and the colony) of females (red) and males (blue). Distribution of all variables are displayed in the form of one-dimensional density plots. Two-dimensional scatterplots with ellipses represent trophic niches in two-dimensional perspectives of two variables. Boxplots show median (band inside the box), the first (25%) and third (75%) quartile (box), the lowest and the highest values within 1.5 interquartile range (whiskers), and outliers (dots). Guillemot’s graphic by K.C.

The realized 3-dimensional foraging habitat niches (reflected by SST, DEPTH and COLDIST) of the GPS-tracked females were significantly broader than those of males (Wilcoxon test; V = 2,001,000; *P* < 0.0001) (Fig. 5; Table 4). Thus, foraging habitat niche overlap was lower for females vs. males (76–88%) compared to males vs. females (83–92%) (Table 4).

**Table 4:** Foraging habitat niche breadth of the chick rearing GPS-tracked female and male Brünnich’s Guillemots breeding in Hornsund (SW Spitsbergen) and inter-sex overlap (95% and 99% niche region sizes).

Variable		Female	Male
Foraging niche breadth	Mean	121,440.43	103,442.34
	SE	3,940.20	2,402.70
Foraging niche breadth overlap 95%	**Female vs**	-	75.62
	**Male vs**	82.52	-
Foraging niche breadth overlap 99%	**Female vs**	-	87.62
	**Male vs**	92.01	-

SE – standard error.

Distribution of COLDIST exhibited three peaks at approx. 10, 40, and 70 km in females and two peaks at approx. 10 and 50–70 km in males. Distribution of SST in foraging locations was characterised by three peaks at approx. 5, 7, and 8 °C in females and at approx. 4, 5.5 and 7 °C in males. Distribution of DEPTH in females showed that they used areas of greater depths (< 500 m), with a single strong peak for approx. 200 m. Males foraged more frequently at the shelf zone (approx. 200–0 m) and generally avoided greater depths beyond the shelf (Fig. 5).

## Discussion

In this study, combining GPS-tracking and remote sensing data, we found that foraging trip characteristics were generally similar between sexes (duration, maximal and total distances covered during trips, home and core range areas, number of trips and dives per day, colony attendance). However, we found significant differences in the foraging locations characteristics, diving pattern number of trips per sex, and foraging habitat niche breadth. Females foraging locations were located further from the colony, compared to males. Environmental conditions at the foraging locations of GPS-tracked individuals differed significantly between sexes. Females foraged in warmer water masses, in areas characterised by greater depths, compared to males. Broader foraging habitat niches of females indicate that they utilised broader range of microhabitats than males. As we expected, females were more strongly affected by the temporal food resource depletion effect than males. This was indicated by more frequent dives and more rapid increase in the distance between foraging locations and the colony.

In contrast to our expectation, we did not find sex differences in foraging trip characteristics. However, foraging locations of females were situated on average significantly further from the colony compared to the ones utilised by males. Sex differences in distances from the colony to foraging locations might indicate different foraging strategies [e.g., 5]. Intersexual variation in some foraging characteristics has been reported for the Low Arctic guillemot populations. On the Gannet Islands off the coast of Labrador (Low Arctic) male guillemots perform longer trips and very likely deeper dives than females^21,23^. However, our findings from Spitsbergen indicate the opposite pattern with females foraging further from the colony and utilising greater depths. Female guillemots from the Coats Island colony (Low Arctic Canada) also have been reported to perform longer trips (in terms of maximum, total and average distance)^84^. The differences between our findings and those from mentioned Low Arctic populations could be due to the more pronounced diurnal cycle there. This results in a shorter period of time during the day allowing birds to forage. In the High Arctic, due to the 24-h polar day, there are no such time limits for foraging. This could be the reason behind no sex differences in trips properties, detected in our study. Absence of sex differences in foraging behaviour in guillemots have been previously reported from the High Arctic during polar summer. According to^24^, no sex-specific spatial foraging strategy (diet and habitat use) was identified in the guillemot population from Greenland. However, some temporal intersexual patterns were observed during the diurnal cycle, suggesting the influence of social or ecological mechanisms^24^. Still, more data from different colonies, latitudes and years with various food availability are needed to understand whether the foraging behaviour is environmental condition dependent.

We found support for the energetic constraint hypothesis predicting sex-specific temporal changes in energy allocation into self-maintenance and chick provisioning^12^. In contrast to other studies^5,12,23^ we did not find differences in chick provisioning (frequency of feeding visits reflected by the number of foraging trips per day). However, given similar duration and distance and frequency of foraging trips in both sexes, the number of dives per trip may serve as a proxy of foraging intensity. Increasing number of dives per trip with progress of the chick-rearing period may be interpreted as balanced investments on self-maintenance and chick provisioning at the early phase of chick rearing and more chick-provisioning biased investments at the end of this period. The opposite temporal trend in dives number in males may be interpreted as balanced investments at the beginning of the chick rearing period and lower investment in chick provisioning at the end of the nesting period, as suggested by other studies^6,12,22^ in anticipation of the post-fledging duties (at sea male only care of offspring).

The studied GPS-tracked guillemots generally foraged in cold waters with low primary productivity at rather shallow areas with flat seabed (low slope). Range of SST in foraging locations (4–8 °C) corresponds to the thermal range of guillemot’s preferred cold-water prey (e.g., polar cod) occurrence. Both juvenile and adult polar cod are common in Svalbard during summer^85^. The majority of juveniles (approx. 6 months old) polar cod in the Barents Sea have been found at water masses with temperatures ranging between 2.0 and 5.5 °C, while adult fish from -5 °C to 5 °C^86–88^. In this study, male guillemots were observed to forage at locations with more suitable/optimal foraging conditions (colder, more productive, shallow water zone with flatter sea floor) compared to females. Observed intersexual differences in characteristic of utilised foraging locations could be related to, e.g., competitive exclusion, different capabilities of sexes to exploit specific prey types (e.g., due to differences in diving capacity between sexes), or perhaps our findings could represent risk-partitioning between sexes while foraging (described by^5^). Male guillemots are often more engaged in protection of offspring against predators^20^ and take exclusive care of the fledgling at the sea after leaving the colony^5–7^. Thus, they might adopt a risk-averse strategy constantly providing optimal amount of food/care to their offspring left at the colony. Males while feeding closer to the colony on the shelf, likely could forage on invertebrates (very likely benthic taxa). Finding and obtaining of such prey is less time-consuming compared to high-energy prey (fish) delivered by females^89,90^. The more ‘risk-prone’ strategy of females may also allow them to forage in more diverse habitats while foraging, i.e., local underwater currents, eddies, or cold underwater prey abundant refugia^91^. Guillemots may also utilise prey provided by highly productive thermal fronts at the shelf break^17,38^ (Fig. 2 g-h).

Indeed, the foraging habitat niches were broader in females foraging across a wider range of temperature and depth zones than males. Use of the narrower habitat niche focusing on less demanding but easier to catch prey may provide males with enough energy for both self-maintenance and securing chicks needs at relatively low foraging costs. Searching for energy-rich prey may be optimal strategy for females at the beginning of the chick rearing period when they may both invest in self-maintenance (replenishing their reserves after initial investments) and provide chicks with good quality food. At later phases of chick growth when males provisioning is lower (expressed by lower number of dives per trip in our study and lower provisioning rate in other studies [e.g., 22]). Females may invest more heavily in chick provisioning, potentially at a short‑term energetic cost, but because they conclude their parental duties earlier than males, they have an opportunity to replenish their reserves sooner. It illustrates how investments in self-maintenance and chick provisioning may be balanced thanks to sex-specific temporal changes in resources allocation and risk-partitioning.

Temporal changes in numbers of dives and distance between foraging locations and the colony may also be driven by temporal food depletion halo detected in this study and also in another study from the same colony including two breeding seasons including the one studied here^25^. However, we do not know which type of prey is responsible for this effect (invertebrates or fish) due to the scarcity of studies of temporal variation in prey availability in the studied area.

We are aware of some limitations of our study. First, our result may be affected by potential negative effect of GPS-logger on birds’ behaviour (though the devices were well below the recommended weight threshold), small sample size, and lack of diet analysis. Second, we performed this study only during one year with specific environmental conditions which poorly reflect the high inter-annual variability of conditions at sea in High Arctic. Therefore, we encourage future studies to include a wider time span (several years) and multiple study sites, in order to gain a better understanding of birds’ behavioural responses to environmental conditions.

## Conclusions

Here we report poorly investigated intersexual variability of foraging ecology of guillemots in the High Arctic colony during the chick-rearing period. We found no sex differences in trips characteristics. This result can be explained by stable light conditions during the polar day and/or interannual differences in food availability, as some differences between sexes were noted from lower latitudes for this species. Still, females foraged further from the colony in less optimal foraging locations (areas of greater depths with warmer water masses) than males. Females also exploited wider range of habitats especially ones with diversified surface temperature and sea depth, while males preferred habitats with lower range of environmental conditions. Our results suggest different foraging techniques or feeding on different prey items in both sexes. We found that with progression of the chick-rearing period, temporal prey depletion affects both sexes. At the late chick-rearing phase, birds (females more rapidly than males) started to forage in further locations, in water with suboptimal conditions and dove more frequently. Our study illustrates how investments in self-maintenance and chick provisioning may be balanced thanks to sex-specific temporal changes in resources allocation and risk-partitioning. Our findings provide valuable insights into the interplay between sex, foraging strategy, and reproductive roles in monomorphic seabirds.

## Data Availability

The dataset supporting this study is available online at OSF repository: https://osf.io/qp7mj
